# Annual acknowledgement of manuscript reviewers

**DOI:** 10.1186/1471-2342-13-8

**Published:** 2013-04-23

**Authors:** Adrian Aldcroft

**Affiliations:** 1 BioMed Central, Floor 6 236 Gray's Inn Road, London, WC1X 8HB, UK

## Abstract

**Contributing reviewers:**

The editors of *BMC Medical Imaging* would like to thank all our reviewers who have contributed to the journal in Volume 12 (2012).

## 

Marcus Abboud

USA

Jafri M. Abdullah

Malaysia

Takashi Abe

USA

Begoña Acha

Spain

Ryan Aleong

USA

Esam Alhamad

Saudi Arabia

Estanislao Arana

Spain

Ulas Bagci

USA

Sebastian Baldi

Argentina

Perry Binder

USA

Gilles Capellier

France

Settimo Caruso

Italy

Donato Cascio

Italy

Pietro Cerveri

Italy

Davinder Chadha

India

Yu Fan Cheng

Taiwan

Yong-Pil Cho

Korea South

Soon-Chul Choi

Korea South

Francesco Ciompi

Spain

Jennifer Collinger

USA

Giuseppe Coppini

Italy

Cristiana Corsi

Italy

Pavel Crystal

Canada

Sarada Dakua

India

Frederik De Keyzer

Belgium

Giorgio De Nunzio

Italy

Andrew Degnan

USA

Iwan Dobbe

Netherlands

Paul Edison

United Kingdom

Ayman El-Baz

USA

Xiaobing Fan

USA

Ales Fidler

Slovenia

Rosemarie Forstner

Austria

Dimitrios Fotiadis

Greece

Robert Fox

USA

Giovanni Frisoni

Italy

Claus Fristrup

Denmark

Dongwei Gao

USA

Bernhard Gerber

Belgium

Georgi Gerganov

Bulgaria

Rosalind Given-Wilson

United Kingdom

Lode Goethals

Belgium

Patrick Goodwill

USA

Marcel Greuter

Netherlands

Boris Gutman

USA

Marianne Haapea

Finland

Takaomi Hanaoka

Japan

Yasunobu Hayabuchi

Japan

Cameron Holloway

Australia

Yang Hongqin

Christmas Island

Satoshi Honjo

Japan

Jongkai Hsiao

Taiwan

Tarique Hussain

United Kingdom

Ula Hwang

USA

Theo Jäger

Germany

Rachid Jennane

France

Jingfeng Jiang

USA

Jukka Jolkkonen

Finland

Mehmet K¿L¿Nç

Turkey

Arvid Kamps

Netherlands

Bong Joo Kang

Korea South

Porkumaran Karantharaj

India

Stijn Keereweer

Netherlands

Nefati Kiylioglu

Turkey

Joshua Klein

USA

Juha Koskenvuo

USA

Jason Koutcher

USA

Frithjof Kruggel

USA

Lewis Kuller

USA

Thomas Kwee

Netherlands

Hans Lamecker

Germany

Eric Lespessailles

France

Jennifer Li

USA

Timo Liimatainen

Finland

Myong Cheol Lim

Korea South

Wenzhong Liu

China

Vanathi M

India

Anant Madabhushi

USA

Ferdinando Maggioni

Italy

Sokratis Makrogiannis

USA

Jorge Marquez

Mexico

Abraham Martin

Spain

Fadi Matta

USA

Sven Mieog

Netherlands

Kyung-San Min

Korea South

Bernd Mueller-Bierl

Belgium

Hakan Mutlu

Turkey

Takeshi Nakaura

Japan

John Nance

USA

Uwe Nixdorff

Germany

Antoine Nordez

France

David Norris

Netherlands

Robert Ogg

USA

Tick Hui Oh

Malaysia

Satoshi Okayama

Japan

Olivier Outteryck

France

Serhat Ozekes

Turkey

Gustavo Pacheco

USA

Ramasamy Paulmurugan

USA

Franjo Pernus

Slovenia

Franco Pessana

Argentina

Päivi Liisa Piirilä

Finland

Jiantao Pu

USA

Alexander Rauscher

Canada

Luca Saba

Italy

Balasrinivasa Sajja

USA

Vera Schrauwen-Hinderling

Netherlands

Claudia Sciallero

Italy

Lubdha Shah

USA

Feng Shi

USA

Yonghong Shi

China

Atul Shinagare

USA

Karuppanagounder Somasundaram

India

Christof M Sommer

Germany

Jacob Sosna

Israel

Bruce Shawn Spottiswoode

South Africa

Patrick Stroman

Canada

Songyuan Tang

China

George Themelis

Germany

Niels Thomsen

Sweden

Catalina Tobon Gomez

Spain

Giampaolo Tomasi

United Kingdom

Yunjie Tong

USA

Robert Toth

USA

Moez Trigui

Tunisia

Ioannis Tsalafoutas

Greece

Gooitzen Van Dam

Netherlands

Marianna Vlychou

Greece

Jane Wang

Taiwan

Ulrich Weber

Switzerland

Gerlig Widmann

Austria

Einar Wilder-Smith

Singapore

Zhong Xue

USA

Lydia Zablotska

USA

Philippe Zerbib

France

Liang Zhan

USA

Nanyin Zhang

USA

## Pre-publication history

The pre-publication history for this paper can be accessed here:

http://www.biomedcentral.com/1471-2342/13/8/prepub

